# Comparative proteomics of *Listeria monocytogenes* strains of food and clinical origin reveals strain-specific adaptation mechanisms

**DOI:** 10.3389/fmicb.2025.1640990

**Published:** 2025-08-26

**Authors:** Ivanka Krasteva, Maria Schirone, Chiara Di Pancrazio, Francesco Manocchia, Federica D'Onofrio, Marta Maggetti, Fabrizia Perletta, Francesco Pomilio, Gianfranco Bruno, Marina Torresi, Gabriella Centorotola, Antonello Paparella, Flavio Sacchini, Nicola D'Alterio, Mirella Luciani

**Affiliations:** ^1^Istituto Zooprofilattico Sperimentale dell'Abruzzo e del Molise “G. Caporale”, Teramo, Italy; ^2^Department of Bioscience and Technology for Food, Agriculture and Environment, University of Teramo, Teramo, Italy; ^3^Ospedale “San Salvatore” – L'Aquila – ASL1 Abruzzo, L'Aquila, Italy

**Keywords:** foodborne pathogen, comparative proteomics, environmental adaptation, immunogenic proteins, host-pathogen interaction, ready-to eat food, *Listeria monocytogenes*

## Abstract

*Listeria monocytogenes* is a foodborne pathogen capable of surviving in diverse environments, including food-processing settings and the human host. This study compared the proteomic profiles of two *Listeria monocytogenes* strains grown at 37 °C to simulate host-associated conditions: a hypovirulent, food-derived strain and a hypervirulent strain isolated from a human clinical sample. This approach enabled the identification of temperature-induced changes in virulence factors, providing valuable insights into molecular determinants of pathogenicity and potential intervention strategies. Mass spectrometry identified 954 proteins, 642 of which were predicted to be immunogenic. Among these, 128 were unique to the food-derived strain (F), and 29 were specific to the clinical strain (H). Functional analysis revealed that F-specific proteins were primarily involved in terpenoid backbone biosynthesis and the production of secondary metabolites, processes associated with membrane integrity, stress resistance, and metabolic adaptation. In contrast, H-specific proteins were related to acid resistance and bacteriophage-associated functions. Although the number of H-specific immunogenic proteins was insufficient for statistically significant enrichment analysis, six highly interconnected proteins were identified. These results suggest that *L. monocytogenes* undergoes targeted proteomic remodeling under host-mimicking conditions, facilitating its transition from a food contaminant to invasive pathogen. The identification of immunogenic, strain-specific proteins enhances our understanding of bacterial adaptation and virulence, with important implications for diagnostics, surveillance, and targeted mitigation efforts.

## 1 Introduction

Listeriosis, a foodborne illness caused by *Listeria monocytogenes*, occurs in two forms: (i) non–invasive, in which bacteria remain confined to the intestinal tract and cause flu-like symptoms in healthy individuals, and (ii) invasive, where the pathogen spreads beyond the intestines, potentially leading to severe outcomes—particularly in vulnerable populations, such as pregnant women, the elderly, and immunocompromised individuals—(Koopmans et al., 2022). Given the aging population in Europe (currently 21.3%) (Eurostat, 2025), and the increasing prevalence of chronic diseases, the risk of severe listeriosis is expected to rise (EFSA BIOHAZ Panel, 2020).

In the United States, listeriosis ranks as the third leading cause of death from foodborne diseases, with approximately 260 fatalities annually. Most infections require hospitalization, and the case-fatality rate is around 20% (CDC. Centers for Disease Control and Prevention, 2024). Historically, meat and poultry—especially ready-to-eat (RTE) products, have been the main sources of outbreaks, accounting for nearly 46% of cases between 1998 and 2007. More recent data show a 2.9% prevalence of *L. monocytogenes* in deli meats and 0.3% in retail store samples. Due to their widespread consumption and extended shelf life, RTE foods pose a significant contamination risk, especially since *L*. *monocytogenes* can proliferate over time. Deli meats sliced in-store are five times more likely to cause listeriosis compared to those pre-sliced in the manufacturing plants, largely due to cross-contamination in the retail environment (Forauer et al., 2021). In response, the USDA implemented a zero-tolerance policy for *L*. *monocytogenes* in RTE meats in 2003 (Belias et al., 2024), followed by the introduction of post-processing treatments and antimicrobial agents. However, by 2008, fresh produce overtook meat as the leading source of outbreaks, driven by increased demand for fresh and convenient foods. The Food Safety Modernization Act (FSMA) introduced stricter controls to manage contamination during production and distribution. Moreover, outbreaks linked to soft, unpasteurized cheeses prompted the adoption of non-thermal technologies— such as high-pressure processing and ultraviolet light—to reduce microbial risks (Su et al., 2024).

In the European Union (EU), invasive listeriosis is notifiable under Directive 2003/99/EC. According to the EFSA 2023 report (EFSA and ECDC (European Food Safety Authority and European Centre for Disease Prevention and Control), 2024), 2,952 confirmed cases were recorded, with a notification rate of 0.66 cases per 100,000 population, marking a 5.8% increase compared to 2022 (0.63 per 100,000 population). This outcome represents the highest number of cases since EU-level monitoring began in 2007, placing listeriosis as the fifth most frequently reported zoonosis. The case-fatality rate fluctuated over the years, reaching 18.1% in 2022, higher than 13.7% in 2021 and 13.0% in 2020. However, the recently observed decline was likely due to reduced reporting during the COVID-19 pandemic (EFSA and ECDC (European Food Safety Authority and European Centre for Disease Prevention and Control), 2021, 2022, 2023). Between 2014 and 2023, over 150 outbreaks occurred in the EU, with a record 35 in 2022. Germany (7), Denmark (6), Austria (5), Italy (4), with Spain, Finland, and Ireland each reporting 3 outbreaks were the most affected countries (EFSA and ECDC (European Food Safety Authority and European Centre for Disease Prevention and Control), 2023). Despite ongoing mitigation efforts, recalls of contaminated products remain frequent. From 2020 to 2024, 615 notifications were issued via the European Commission's Rapid Alert System for Food and Feed (RASFF), including 133 alerts for milk products, 86 for fish, and 93 for meat (excluding poultry meat), reflecting ongoing safety concerns (RASFF Window). Contaminated RTE foods, such as cold smoked salmon, meat products, and dairy products, were the primary sources of infection. In these products, *L. monocytogenes* exceeded regulatory limits in 0.11% to 0.78% of samples, with fermented sausages showing the highest contamination levels (EFSA and ECDC (European Food Safety Authority and European Centre for Disease Prevention and Control), 2024). Due to the absence of further cooking before consumption, RTE foods are particularly vulnerable to contamination and necessitate a strict control. To address this risk, Regulation (EU) No. 2024/2895, amending Regulation (EC) No. 2073/2005, introduced enhanced microbiological criteria for *L. monocytogenes* in RTE foods that support bacterial growth. Effective from December 2024 and fully applicable by July 1^st^, 2026, the Regulation requires that *L. monocytogenes* shall be undetectable in 25 g of RTE food, unless the manufacturer can demonstrate to the competent authority that levels remain below 100 CFU/g throughout the product's shelf life. Previously, this requirement applied only while the product was under the responsibility of the food business operator. The revised rule now extends this obligation throughout the entire shelf life, marking a proactive shift toward consumer protection.

*L. monocytogenes* is a heterogeneous species, subdivided into different clonal complexes (CCs) according to multilocus sequence typing (MLST) results. Three categories of prevalent clones were identified: hypervirulent infection-associated clones (CC1, CC2, CC4, and CC6), hypovirulent food-associated clones (CC9 and CC121) and intermediate clones (CC8-16, CC5, CC3, CC37, CC155, and CC18) (Maury et al., 2016). While hypervirulent clones, particularly CC1, are associated with human clinical cases and dairy products, hypovirulent ones, especially CC9, are strongly associated with meat and meat products (Maury et al., 2019).

To control *L. monocytogenes* throughout the food chain, the One Health approach is essential as transmission can occur via contaminated food, water, or contact with infected animals, particularly ruminants, which serve as reservoirs. Although, direct transmission from animals to humans is rare, animals significantly contribute to environmental contamination, which is perpetuated through fecal matter and wastewater (Filipello et al., 2020; Tsitsos et al., 2025). Once introduced into the processing facilities, *L. monocytogenes* is difficult to eliminate due to its capacity to form biofilms on biotic or abiotic surfaces, survive a wide range of temperatures (from−2°C to 45°C), resist antimicrobial agents, and tolerate acidic, high-salt, and low-oxygen conditions (Belias et al., 2022; Murugesan et al., 2015). To cope with these stress conditions, *L. monocytogenes* strains, mainly the hypovirulent CC9 and CC121 clones, typically carry several genomic resistance factors for environmental adaptation (Maury et al., 2019). Based on its behavior in food-processing environments *L. monocytogenes* can be classified into three categories: (i) persistent strains, which survives over long periods despite cleaning and disinfection; (ii) transient strains, which are introduced but successfully eliminated, and (iii) persistent transient strains, which are continuously introduced through raw materials or surrounding environments (Ferreira et al., 2014). The exact mechanisms behind persistence remain unclear, as some studies have described complex environmental interactions (Taylor and Sasiewicz, 2019), while others have blamed poor sanitation or design flaws in food facilities (Carpentier and Cerf, 2011). A key factor in persistence is the formation of biofilms, which are notably more resistant to standard cleaning methods (Borucki et al., 2003; Pan et al., 2006). Since retail environments represent a critical point of contamination for fully virulent *L. monocytogenes* strains in RTE foods, identifying transmission routes and reducing persistence is vital to minimizing infection risks. Differences among strains significantly influences the microbial ability to adapt and persist for extended periods in environmental niches, as well as the potential to cause disease (Kovacevic et al., 2016). Therefore, understanding the mechanisms that allow *L. monocytogenes* to survive in diverse environments is crucial for elucidating its pathogenic potential.

Although proteomic studies on *L. monocytogenes* have been conducted, this investigation provides a focused comparison between two well-characterized reference strains at opposite ends of the virulence spectrum. Specifically, we analyzed CC1 (hypervirulent) and CC9 (hypovirulent), which belong to distinct genetic lineages (I and II, respectively) and are phylogenetically distant. This comparison is biologically relevant: CC1 strains are predominantly associated with invasive human infections, whereas CC9 strains are commonly isolated from food-processing environments to exhibit enhanced environmental persistence. To explore whether their proteomic profiles reflect differences in ecological adaptation and pathogenic potential, both strains were cultivated under identical and controlled growth conditions (37°C). Particular emphasis was given to immunogenic and surface-associated proteins potentially involved in host-pathogen interactions and environmental stress responses.

The objective of this study is to conduct a comprehensive proteomic analysis of a clinical CC1 isolate and a food-derived CC9 isolate, aiming to identify molecular determinants linked to virulence, stress tolerance, and environmental persistence. While similar comparative studies have been reported, our dataset reveals novel immunogenic surface proteins and strain-specific metabolic regulators not previously described in this context. This framework offers valuable insights into the molecular mechanisms underlying strain-specific differences in pathogenicity and survival. Understanding these differences is essential for improving risk assessment and informing the development of targeted interventions in food safety and public health.

## 2 Materials and methods

### 2.1 Bacterial strain and growth conditions

Two *L. monocytogenes* strains, previously characterized by whole genome sequencing approach, were provided by the Italian National Reference Laboratory for *L. monocytogenes* (LNR-*Lm*) at the Istituto Zooprofilattico Sperimentale dell'Abruzzo e del Molise (IZSAM), Teramo, Italy. The former (F) was a CC9 isolated from pork sausage while the latter (H) was a CC1 isolated from blood of infected patient.

The two *L. monocytogenes* genomes were deposited at DDBJ/ENA/Gen Bank under the Bio Project PRJNA1267817. All the details related to *L. monocytogenes* strains considered in this study are reported in Table 1.

**Table 1:** Strains identification of two *L. monocytogenes* isolates and related information on isolation source, clonal complex, the main virulence and stress resistance factors, obtained *in silico*, and the BioSample accession numbers.

**Strain identification**	**Isolation source**	**Clonal Complex**	**Main virulence factors**	**Main stress resistance factors**	**Biosample accession number**
H	Human	CC1	Full-lengthed *inlA*, complete LIPI-1 and LIPI-3	Complete SSI-1	SAMN48730946
F	Pork sausage	CC9	Truncated *inlA* and complete LIPI-1	Incomplete SSI-1 (lmo0447)	SAMN48730945

*L. monocytogenes* was cultivated in Brain Heart Infusion (BHI) broth (Oxoid, Thermo Fisher Scientific, Rodano, Italy) supplemented with 0.5% NaCl (pH 7.0), incubated at 37°C. The bacterial culture was grown overnight in BHI medium, diluted 1:100 in fresh BHI, and cultivated under constant shaking until it reached the exponential growth phase (OD_600_ = 0.6).

Cells were harvested by centrifugation (Eppendorf, Hamburg, Germany) at 4,500 × g for 15 min at 4°C, washed three times with sterile ice-cold 0.01 M phosphate-buffered saline (PBS, pH 7.0), and kept on ice. The resulting pellet was stored at−80°C until use. Three biological replicates were performed.

### 2.2 Protein extraction and tryptic digestion

Proteins were extracted using CelLytic B Cell Lysis Reagent and CelLytic IB Inclusion Body Solubilization Reagent (Sigma-Aldrich, Saint Louis, Missouri) according to the manufacturer's instructions. After enzymatic extraction, cell debris was removed by centrifugation, and the supernatants were collected for further analysis. Protein concentrations were determined using the Bradford assay (Bio-Rad, Hercules, California).

For tryptic digestion, 30 μg of protein extract from each sample were processed using the filter-aided sample preparation (FASP) method. Proteins were alkylated with 50 mm iodoacetamide and buffered with 50 mm ammonium bicarbonate. Trypsin was added at a substrate-to-enzyme ratio of 50:1 (v/v), and digestion was carried out overnight at 37°C. The reaction was quenched by acidification with 10% formic acid. The resulting peptide mixtures were desalted using a Pierce C18 Spin column (Thermo Fisher Scientific) before being analyzed by mass spectrometry.

### 2.3 Mass spectrometry analysis

Five microliters (μL) of extracted peptides from each sample were analyzed in triplicate using Liquid Chromatography-Tandem Mass Spectrometry (LC-MS/MS) on an Easy-nLC 1,200 nano system (Thermo Fisher Scientific, Waltham, Massachussets) coupled to an Orbitrap Q-Exactive mass spectrometer (Thermo Fisher Scientific). Peptides were first loaded onto a PepMap pre-column (75 μm I.D., 200 mm L., Thermo Fisher Scientific) and subsequently separated using an EASY-Spray C18 analytical column (75 μm I.D., 200 mm L., Thermo Fisher Scientific). A 97-min chromatographic gradient was applied for separation.

The mass spectrometer operated at a resolution of 70,000 in full scan mode, utilizing Data-Dependent Acquisition (DDA) with a “top 12” approach for shotgun proteomics. Raw data were processed using Proteome Discoverer (version 2.5, Thermo Fisher Scientific) with database searches against the *L. monocytogenes* 1/2a strain ATCC BAA-679/EGD-e reference proteome retrieved from the UniProt database. Search parameters included a parent ion mass tolerance of 10 ppm, and a fragment ion mass tolerance of 0.02 Da. Trypsin was specified as the cleavage enzyme, with carbamidomethylation of cysteine set as a fixed modification, while methionine oxidation and N-terminal protein acetylation were considered variable modifications.

Only proteins with high-confidence identifications according to a false discovery rate (FDR) threshold, supported by at least two peptides and classified as master proteins, were considered reliable. For downstream analysis, only proteins identified in at least two out of three biological replicates were included.

### 2.4 Bioinformatic analysis

The data underwent bioinformatics analysis for protein selection. To evaluate the subcellular localization of the proteins, the following software tools were used: LipoP 1.0 server (Juncker et al., 2003) for predicting signal peptides of lipoproteins in Gram-positive bacteria, the TMHMM server version 2.0 for transmembrane helix prediction (Krogh et al., 2001**)**, and the SignalP 5.0 server for signal peptide identification (Petersen et al., 2011; Hallgren et al., 2022). Subcellular localization was predicted using PSORTb version 3.0.2 (Yu et al., 2010) and the Cello server version 2.5 (Yu et al., 2006, 2009).

To identify potential immunogenic candidates, VaxiJen version 2.0 was employed (Doytchinova and Flower, 2007). This software is an alignment-independent server used to predict antigenic properties and physicochemical characteristics of proteins. Proteins with an adhesion score above 0.4 were considered potential antigens.

Protein interaction networks and functional enrichment analyses, including Gene Ontology (GO) classification, were conducted using the STRING database (v12.0; https://string-db.org/), with *L. monocytogenes* as the reference organism and a minimum confidence score of 0.900. All interaction sources were enabled, and only query proteins were displayed. Visualization was performed in interactive SVG format with 3D bubble design, and disconnected nodes were hidden. Protein taxonomy and annotation were analyzed using the UniProt database (https://www.uniprot.org/).

## 3 Results

### 3.1 Mass spectrometry (nLC-ESI-MS/MS)

**Figure 1:**
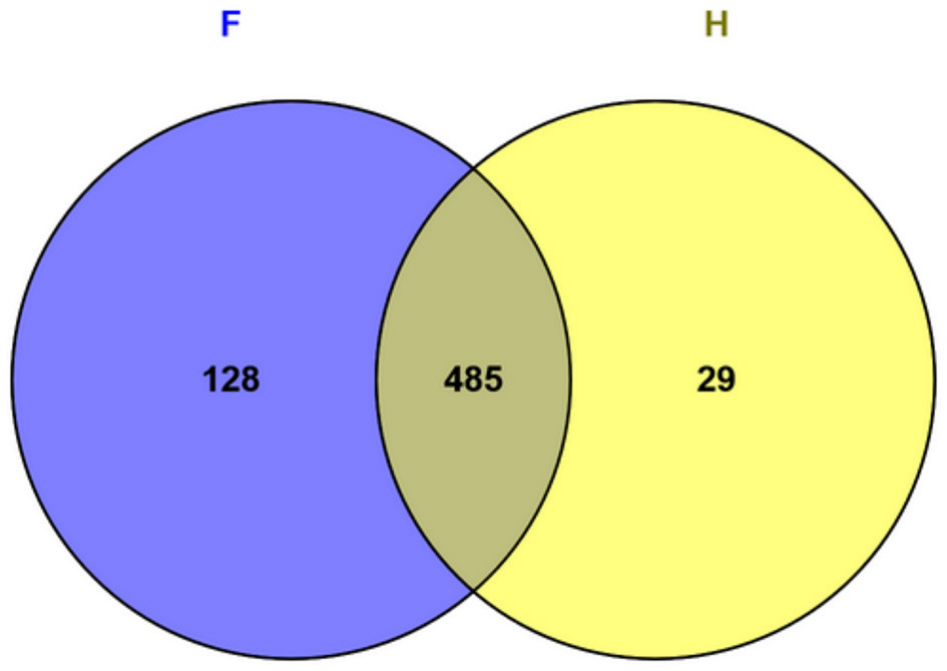
Venn diagram showing the number and percentage of immunogenic proteins identified in the hypovirulent (F) and hypervirulent (H) strains.

A total of 954 proteins were identified through mass spectrometry analysis (Supplementary Table 1). Subcellular localization was predicted using the Cello Server v2.5, LipoP 1.0 Server, TMHMM Server v2.0, SignalP 5.0 Server, and PSORTb v3.0.2. Among these, 766 proteins were classified as cytosolic, while 188 were identified as non-cytosolic, as detailed in Supplementary Table 2. To identify potential immunogenic candidates, all identified proteins were further analyzed using the VaxiJen v2.0 tool. This analysis yielded 642 putative immunogenic proteins, 128 of which were uniquely expressed in the food-derived strain (F), whereas 29 were exclusive to the human-derived strain (H) (Supplementary Table 3). The remaining 485 proteins were shared between the two experimental groups (Figure 1).

### 3.2 Bioinformatics analysis

**Figure 2:**
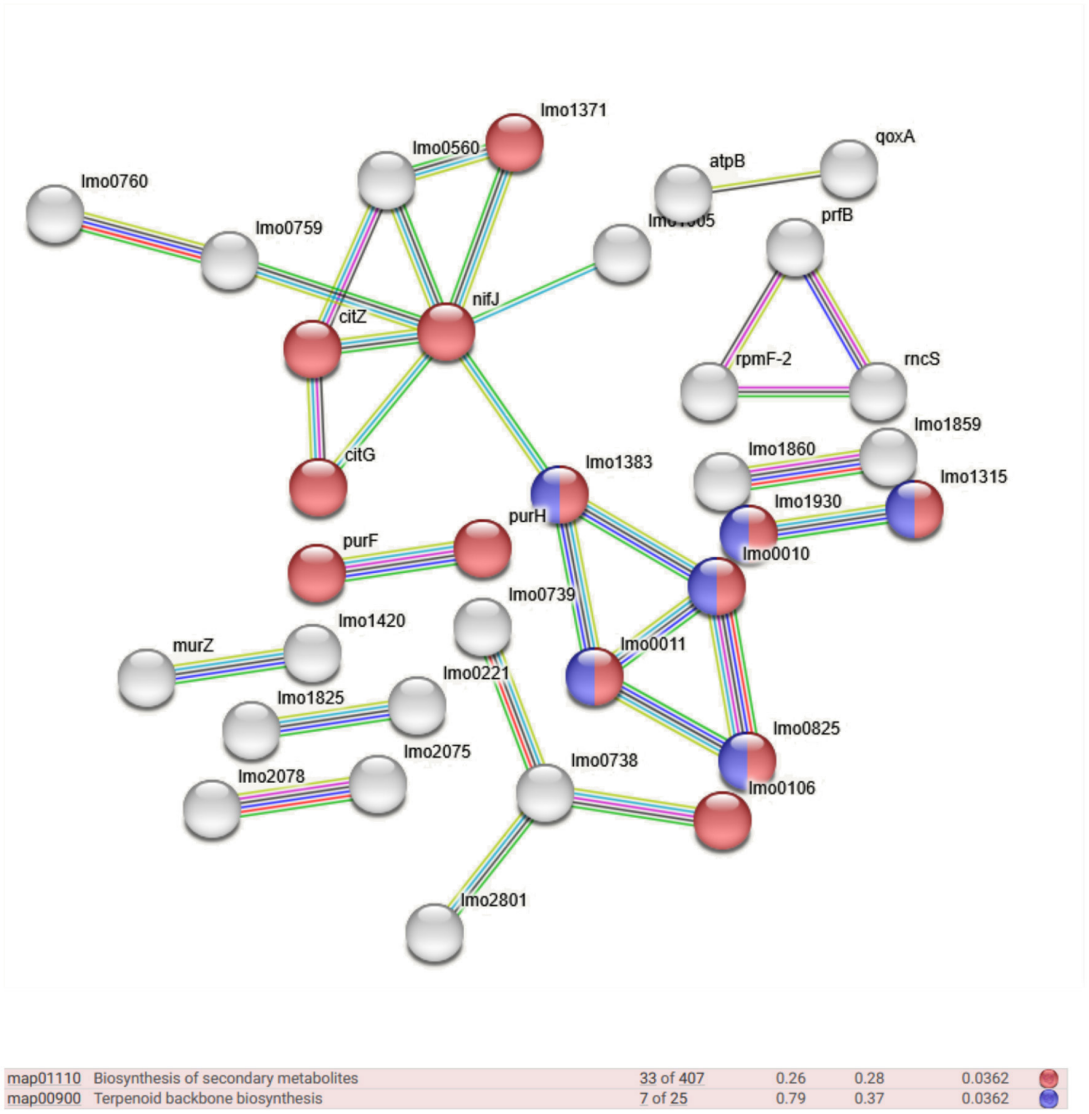
STRING high-resolution bitmap representing the network of different biological processes in which the uniquely expressed proteins from the F dataset are involved.

Qualitative networks derived from Gene Ontology (GO) analysis were visualized and examined using the STRING server v.12.0 to investigate the molecular functions of immunogenic proteins expressed in the F and H datasets. The STRING server gathers and analyzes physical and functional protein-protein interactions, integrating data from multiple sources and critically scoring interactions based on hierarchical orthology. As shown in Figure 2, a subset of the 128 proteins uniquely expressed in the food-derived dataset (F) was associated with several key biological processes, including terpenoid backbone biosynthesis (7 proteins, FDR 0.0362) and biosynthesis of secondary metabolites (33 proteins, FDR 0.0362). In contrast, the 29 unique immunogenic proteins expressed in the human-derived dataset (H) were not sufficient to identify statistically significant enriched biological processes. However, six interconnected proteins were identified through STRING network analysis (Table 2), indicating potential functional relevance. Further STRING-based analysis allowed the identification of biological processes involving proteins from both datasets (F to H). Table 3 summarizes the 16 most significantly enriched pathways for each group (613 proteins in the hypovirulent strain – F – and 514 in the hypervirulent strain - H), listed in descending order of statistical significance. Among these, metabolic process, primary metabolic process, and cellular metabolic process showed the highest significance in both datasets. Notably, several pathways were exclusively enriched in the hypovirulent strain (F) including cellular nitrogen compound metabolic process. In contrast, only one pathway —protein metabolic process —was uniquely enriched in the hypervirulent strain (H).

**Table 2:** List of *L. monocytogenes* immunogenic interconnected proteins present in H.

**Gene**	**Protein**	**Protein name**	**MW (kDa)**
*lmo0127*	Q8YAJ7	Similar to protein gp20	19.7
*lmo0122*	Q8YAK2	Similar to phage protein	31.3
*lmo2363*	Q9EYW9	Glutamate decarboxylase beta	53.5
*lmo2362*	Q8Y4S1	Glutamate/gamma-aminobutyrate antiporter	55.1
*lmo2742*	Q8Y3T9	Lmo2742 protein	13.3
*lmo0617*	Q8Y9B5	Lmo0617 protein	17.8

**Table 3:** Main biological processes enriched in F and H samples, described in ascending order of statistical significance using the false discovery rate (FDR) method.

**Go-term**	**Description**	**FDR- strain F**	**FDR- strain H**
*GO:0008152*	Metabolic process	9.51e-20	8.79e-17
*GO:0044238*	Primary metabolic process	1.74e-18	1.50e-17
*GO:0044237*	Cellular metabolic process	9.78e-18	5.74e-15
*GO:0071704*	Organic substance metabolic process	2.04e-16	1.05e-14
*GO:0006807*	Nitrogen compound metabolic process	4.48e-14	5.41e-14
*GO:0043603*	Cellular amide metabolic process	3.71e-14	1.05e-14
*GO:1901564*	Organo-nitrogen compound metabolic process	4.05e-13	5.41e-14
*GO:0009987*	Cellular process	4.05e-13	2.87e-12
*GO:0034641*	Cellular nitrogen compound metabolic process	6.70e-12	-
*GO:0009059*	Macromolecule biosynthetic process	7.39e-12	3.13e-14
*GO:0043604*	Amide biosynthetic process	1.76e-12	3.56e-14
*GO:0006518*	Peptide metabolic process	6.23e−12	3.13e−14
*GO:0043043*	Peptide biosynthetic process	8.51e−12	5.41e−14
*GO:0034645*	Cellular macromolecule biosynthetic process	1.4.9e−11	3.84e−13
*GO:0006412*	Translation	1.40e−11	9.45e−14
*GO:0010467*	Gene expression	1.08e−10	6.89e−12
*GO:0019538*	Protein metabolic process	–	2.42e−12

## 4 Discussion

In this study, two *L. monocytogenes* strains were selected from the LNR-*Lm* collection based on MLST results and genomic factors related to stress resistance and virulence: one CC9 strain derived from a meat product, and one CC1 strain from a clinical sample. Literature reports associate CC9 primarily with food and processing environments (Maury et al., 2019; Guidi et al., 2021), and less frequently with human cases, confirming their hypovirulent characteristics largely linked to *inlA* gene truncations (Maury et al., 2016). Conversely, CC9 strains display enhanced resistance to environmental stresses through various factors (Maury et al., 2019; Centorotola et al., 2021; Guidi et al., 2021), such as the presence of a complete Stress Survival Islet 1 (SSI-1), which contributes to tolerance against different stresses (Gray et al., 2021). On the other hand, CC1 strains are well-known for their association with clinical infections and hypervirulence (Maury et al., 2019). The virulence profile of the CC1 strain is characterized by the presence of accessory *Listeria* Pathogenicity Island (LIPI), including LIPI 3, which encodes listeriolysin S–a hemolytic and cytolytic factor (Vilchis-Rangel et al., 2019; Wiktorczyk-Kapischke et al., 2023)—in addition to the ubiquitous LIPI-1. CC1 strains typically possess a full-length *inlA* gene, essential for host cell invasion (Ireton et al., 2021).

To compare a hypervirulent clinical strain (CC1) with a hypovirulent food-derived strain (CC9), proteomic profiling coupled with bioinformatic analysis was performed on both strains grown at 37 °C. Among the 642 immunogenic proteins identified, 128 were unique to the food-derived strain (F), while 29 were exclusive to the human-derived strain (H). Proteins specific to CC1 included several involved in adaptive responses and virulence mechanisms. Key proteins detected in CC1 included ClpC, an ATP-dependent protease common to both strains, which is involved in protein quality control under stress conditions and known to facilitate intracellular survival and phagosome escape (Karatzas et al., 2003).

The comparative proteomic analysis revealed distinct adaptive strategies, reflecting the ecological niches and virulence potentials of the two strains. Several peptides exclusive to CC1 suggest the activation of host—pathogen interaction mechanisms. Notably, Lmo2363 and Lmo2362, components of the glutamate-dependent acid resistance (GDAR) system, were detected only in the clinical strain. This system helps maintain intracellular pH under acidic stress, such as within phagosomes, thereby supporting intracellular survival and virulence (De Biase and Pennacchietti, 2012). Their presence reinforces the hypothesis of functional adaptation to the intracellular environment.

GO analysis of the human-derived strain (H) showed enrichment in biological processes related to macromolecular biosynthesis and organic nitrogen compound metabolism, consistent with a physiological state favoring active proliferation in host tissues (Smith et al., 2000). While many functional categories were shared between the two strains, the specific proteins involved differed, suggesting functional specialization rather than broad pathway activation. Additionally, the presence of accessory virulence genes encoded by LIPI-3 supports the hypervirulent phenotype of CC1. Although STRING analysis did not highlight significant pathway enrichment overall, six highly interconnected proteins (Q9EYW9, Q8Y4S1, Q8YAJ7, Q8YAK2, Q8Y3T9, and Q8Y9B5) formed a high-confidence interaction cluster (score 0.900). Among them, Lmo2363 (Q9EYW9) and Lmo2362 (Q8Y4S1), part of the GDAR system, regulate intracellular pH and promote acid stress survival. Protein Q8YAJ7, a non-cytosolic component associated with bacteriophage A118 is involved in host recognition and phage assembly. Bielmann et al. (2015) identified this protein, also known as gp20, as a determinant of phage specificity and infectious potential in *L*. *monocytogenes*. Lmo0122 (Q8YAK2), a member of the phage tail protein family, may also contribute to phage-host interactions and serve as a potential phage-binding target.

In contrast, the food-derived strain CC9 (F) exhibited a proteomic profile enriched in proteins involved in terpenoid backbone biosynthesis (e.g., Lmo0825, Lmo0010, Lmo1383, Lmo0011) and secondary metabolite biosynthesis. These proteins are associated with biological processes such as the isoprenoid biosynthetic process, purine nucleoside metabolism, and ribonucleoside bisphosphate metabolism. Similarly, proteins linked to secondary metabolite biosynthesis (Lmo0010, Lmo1383, Lmo1930, Lmo1315 Lmo0011) contribute to broader functions, including lipid biosynthesis and cellular lipid metabolism. These pathways likely play critical roles in membrane integrity, stress resistance, and metabolic adaptation, supporting *L. monocytogenes* persistence in food-associated environments. The annotated proteins (Lmo0825, Lmo0010, Lmo1383, Lmo0011) function as key enzymes in the mevalonate-dependent terpenoid biosynthesis pathway. Begley et al. (2008) confirmed the expression of *lmo0010* (*mvk*) and *lmo0825* (*hmgR*) during host infection, supporting a role for this pathway in intracellular survival. Interestingly, in our study these enzymes were strongly expressed in the food-derived CC9 strain (F), indicating that the mevalonate pathway may also be critical for persistence outside the host. These findings expand the functional relevance of terpenoid biosynthesis beyond virulence, pointing instead to its involvement in stress resistance and membrane homeostasis under harsh environmental conditions such as those found in food-processing settings. Similarly, Diakogiannis et al. (2013) reported changes in the fatty acid composition of *L*. *monocytogenes* under acid and cold stress, highlighting membrane fluidity as a key adaptive trait. Our observation of an enrichment in proteins involved in lipid metabolism and secondary metabolite biosynthesis in CC9 supports this view, indicating that these pathways enhance the strains resilience under environmental challenges such as refrigeration and food-processing conditions. Together, these findings reinforce the hypothesis that CC9 adopts a metabolic strategy centered on long-term survival and stress adaptation rather than active host invasion.

The human-derived strain (H) also showed enrichment in pathways related to macromolecular biosynthesis and organic nitrogen metabolism, consistent with active proliferation in host tissues. Though similar GO categories overlapped between the strains, the specific proteins involved differed, indicating niche-specific adaptations. The clinical strain (H) uniquely expressed key virulence factors such as Act A and Inl B, which are essential for intracellular motility and host cell invasion, as well as other internal in-like proteins aiding immune evasion. It also expressed manganese-dependent superoxide dismutase (MnSOD), which provides protection against oxidative stress during intracellular replication. Shared surface proteins such as Lap B and Auto may play divergent roles in adhesion and colonization depending on the strain.

Overall, the hypervirulent clinical strain exhibited proteomic features aligned with host adaptation and virulence, whereas the hypovirulent food-derived strain showed traits consistent with environmental persistence. These findings highlight distinct evolutionary strategies shaped by the ecological niches of the two strains. The proteins identified in this study could represent promising candidates for functional characterization and potential targets for diagnostic or therapeutic development. Despite identical growth conditions during proteomic analysis, the observed differences likely reflect underlying genomic variations. Whole genome sequencing data are available for both strains and could be integrated into future studies to further elucidate the molecular mechanisms driving phenotypic diversity.

## 5 Conclusion

This study presents a comprehensive proteomic comparison of *L*. *monocytogenes* strains: a hypovirulent isolate from food sources and a hypervirulent strain from human clinical samples, both cultured at 37°C. The differential expression of immunogenic proteins and the identification of distinct functional pathways underscore the bacterium's remarkable ability to adapt to diverse environments and transition between commensal and pathogenic lifestyles. Proteins uniquely expressed in the hypervirulent strain (H, CC1) were associated with acid resistance mechanisms —particularly the GDAR system—and bacteriophage-related functions, which may contribute to enhanced survival and infectivity within the host. In contrast, the food-derived CC9 strain (F) displayed a proteomic profile enriched in proteins involved in terpenoid backbone biosynthesis and secondary metabolite production. These proteins participate in isoprenoid, purine, and lipid biosynthetic processes that support membrane integrity, stress resistance, and metabolic flexibility—traits likely promoting persistence in food-associated environments. Notably, several of these are key enzymes in the mevalonate-dependent terpenoid biosynthesis pathway.

This functional divergence suggests that *L*. *monocytogenes* undergoes targeted proteomic remodeling in response to environmental cues, facilitating its transition from a foodborne contaminant to an invasive pathogen. Although pathway enrichment analysis via STRING was limited, the identification of high-confidence protein-protein interactions and strain-specific immunogenic factors provides novel insights into adaptive strategies underlying virulence and persistence.

Altogether, the proteomic profile of the CC1 strain reflects its nature as a hypervirulent lineage – capable of invading host cells, withstanding intracellular stress, and sustaining replication. In contrast, the CC9 strain appears geared toward surviving in the external environment, with proteins that support stress resistance, nutrient metabolism, and motility. By directly comparing these two strains, this study reveals how *L. monocytogenes* modulates its molecular toolkit according to its ecological niche – whether in a food matrix or within the human host.

The identification of specific immunogenic proteins and enriched pathways provides a valuable foundation for future research into the molecular mechanisms of virulence and persistence, as well as for the development of diagnostic tools and therapeutic interventions.

In conclusion, these findings expand our understanding of *L. monocytogenes* biology and pathophysiology, offering promising avenues for the identification of diagnostic biomarkers, therapeutic targets, and improved strategies for food safety and public health. Further studies are needed to validate the functional roles of the identified proteins and clarify their relevance in the dynamics of infection and environmental survival.

## Data Availability

The datasets presented in this study can be found in online repositories. The names of the repository/repositories and accession number(s) can be found in the article/Supplementary material.
